# Supplementary material for: Acceptance and compliance with micronutrient powder (MNP) among children aged 6–23 months in northern Nigeria
Source: PLOS Glob Public Health. 2022 Oct 17;2(10):e0000961. doi: 10.1371/journal.pgph.0000961 (PMC10022258; doi:10.1371/journal.pgph.0000961)
Supplement: S1 Dataset — (ZIP) [file pgph.0000961.s008.zip › S8_Dataset/Spot Checks.zip!/Spot Checks/020715_K_BK_BK_2_SC.pdf]

SPOT CHECK RECORDING FORM

State: Kebbi LGA: Birnin Kebbi Community: Bakan Yadiya

Sex of index child: Male Age of index child (in months): 14months

Spot Check Information

1. **Date of MNP distribution to household** (circle option below based on LGA name above)**:**

   - **Kebbi:** BirninKebbi&Jega LGA: **June 16**
   - **Kebbi:** Danko-wasagu: **June 17**
   - **Adamawa:** Song, Ganye: **June 18**
   - **Adamawa:**Guyuk **June 19**

2. **Date of Spot Check Observation: 02/07/15**

3. **Number of sachets remaining**(Ask the caregiver to show you and count the specific number of sachets remaining)**:**

   44

4. **Number of sachets that <u>should remain</u> using one MNP sachet per day** (Analyst only)**:**

   45

5. **Number of sachets remaining / number of sachets should be remaining**(Analyst only)**:**

   ______44___ / ____45_____ = ___97.8______ %

Probes

Question 1. You have **XX** MNP sachets remaining. Explain how easy or difficult it was to use the MNP product on a daily basis for your child 6 – 23 months? (Circle one answer)

**Very Difficult**	**Difficult**	**Easy**	**Very Easy**

Question 2. Describe any specific challenges that you faced that made the MNP difficult to use on a daily basis?

Question 3. Describe specific aspects of the MNP product that you or your child enjoyed that made it easier to use on a daily basis?
He was sick when they gave him MNP but when I started giving him I observed that he started improving, so I was encourage that is why I don't forget to give him and now my child that was wasted without strength now is strong and can beat all his age mate

Question 4. What questions do you have about the MNP based on your experience using it?

This MNP is good but I don't know the type of sickness is meant for, please can you tell me the name of the sickness

Question 5. What specific recommendations do you have that we can do as a program to make the MNP easier to use on a daily basis?
Please let it be free so that every child can have access to it because you can see we are poor
